# Covert Network Analysis for Key Player Detection and Event Prediction Using a Hybrid Classifier

**DOI:** 10.1155/2014/615431

**Published:** 2014-07-20

**Authors:** Wasi Haider Butt, M. Usman Akram, Shoab A. Khan, Muhammad Younus Javed

**Affiliations:** Department of Computer Engineering, College of Electrical and Mechanical Engineering, National University of Sciences and Technology, Islamabad 44000, Pakistan

## Abstract

National security has gained vital importance due to increasing number of suspicious and terrorist events across the globe. Use of different subfields of information technology has also gained much attraction of researchers and practitioners to design systems which can detect main members which are actually responsible for such kind of events. In this paper, we present a novel method to predict key players from a covert network by applying a hybrid framework. The proposed system calculates certain centrality measures for each node in the network and then applies novel hybrid classifier for detection of key players. Our system also applies anomaly detection to predict any terrorist activity in order to help law enforcement agencies to destabilize the involved network. As a proof of concept, the proposed framework has been implemented and tested using different case studies including two publicly available datasets and one local network.

## 1. Introduction

From the data published in electronic and print media, it can be clearly concluded that all terrorist events are done by organized terrorist organizations [1]. Members of such organizations cannot operate in isolation and they interact and collaborate with one another in order to coordinate such unlawful events. Law enforcement agencies have information of such members, their affiliations, and communication and other activities of such organizations which are under observation. Krebs introduced “prevention or prosecution” [1]. According to him, the existing methods focus more on prosecution which is of course after the event has occurred. Although there exist very efficient social network analysis (SNA) measures which can be used to find the key players of the identified network, which can be removed to destabilize the network, the thing which lacks is the alarm of the exact time at which those key players should be removed in order to prevent the event.

A social network is a social arrangement made up of a set of social actors such as individuals or organizations and a complex set of the dyadic ties between these actors. The social network perspective provides a clear way of analyzing the structure of whole social entities [2]. SNA is a mathematical method for “connecting the dots,” that is, to analyze nodes and their relationships. SNA allows us to map and measure complex, sometimes covert, human groups and organizations [3]. SNA has been applied in a number of applications in order to explore several interesting features of different sort of social networks especially with the advancement in information and communication technology and availability of social networks in electronic forms.

An important area in SNA is the key player detection. Key player is defined as the most important node in a social network. Centrality is a key theory in the study of social networks in order to study organizational and team behavior. Central individuals control information flow and decision making within a network [4].

Along with key player detection, outlier detection is also important to predict any abnormal activity. Outlier detection deals with detection of patterns from data which do not match expected normal behavior. These anomalous patterns are often known as outliers, anomalies, discordant observations, and so forth in different application domains. Outlier detection is a well-researched area having an immense use in a wide range of applications like fraud detection, insurance, intrusion detection in cyber security, fault detection in security critical systems, military surveillance for enemy activities, and so on.

The paper consists of five sections.  Section 2 describes the existing frameworks related to our proposed one. It also highlights the main contributions which we have made in this field. The detailed explanation of proposed system is given in Section 3.  Section 4 shows the results which are performed to check the validity of proposed method using different networks followed by conclusion and discussion in the last section.

## 2. Related Work

A number of methods for SNA and outlier detection have been proposed. SNA as discussed earlier has been widely used in analyzing social structures. Social structures consist of actors and their relations or interactions. A social structure can be represented in the form of a graph *G*(*V*, *E*) consisting of *V* vertices and *E* edges where each vertex represents a social actor and every edge represents a relationship between two vertices. SNA helps in analyzing and understanding the structural importance of each actor in a network analyzing its relations and their impact throughout the network.

The structural importance of an actor in a network is measured using the centrality. A node is structurally more important to a network if it is relatively more central in a network. Various centrality measures are found in the literature, designed from different perspectives of a network. The most famous are highlighted here. The simplest centrality measure is degree centrality (DC). It uses the number of direct contacts of a node as a pointer of the quality of interconnectedness [4]. Using the adjacency matrix *A* = (*a*_*ij*_), it can be formalized as follows:

(1) $$\begin{array}{c} \text{DC}\left(\upsilon \right)=\overset{n}{\underset{i=1}{\sum }}a_{i\upsilon }. \end{array}$$

Here, DC(*υ*) is the degree centrality of node *υ* in a social network.

For weighted graphs, a variation of DC has been presented by Memon [5]. According to author, for weighted networks, the simplest extension of degree is node strength [6, 7], which is the sum of weights of node's direct ties. Consider

(2) $$\begin{array}{c} \text{WDC}\left(\upsilon \right)=\text{Strength}\left(\upsilon \right)=\overset{n}{\underset{i=1}{\sum }}w_{i\upsilon }, \end{array}$$

where WDC is weighted degree centrality and *w* is the weighted adjacency matrix. The cell *w*_*iυ*_ is greater than 0 if the node *υ* is connected to node *i* and its value represents the weight of the tie.

Another centrality measure based on the idea that nodes with a short distance to other nodes can disseminate information productively through the network is known as closeness centrality (CC) [8]. To calculate the CC of a node *υ*, the distances between the node *υ* and all other nodes of the network are added [9]. By using the inverse value, we achieve that the value of the CC increases when dropping the distance to another node, that is, when improving the integration into network. Formally CC is given by [10]. One has

(3) $$\begin{array}{c} \text{CC}\left(\upsilon \right)=\frac{1}{\sum_{i=1}^{n}d\left(\upsilon ,i\right)}. \end{array}$$

As (3) depicts, the reduction of the distance to at least one other node when adding an additional relationship leads to a smaller value of the denominator in formula, hence increasing the overall values of the measure.

Using betweenness centrality (BC), a node in a network is measured to be well connected if it is positioned on as many of the shortest paths between pairs of other nodes. The fundamental supposition of this centrality measure is that the interaction between two indirectly connected nodes *i* and *j* depends on the nodes between *i* and *j*. The formulation of BC is given by Freeman as [10]

(4) $$\begin{array}{c} \text{BC}\left(\upsilon \right)=\overset{n}{\underset{i=0,i\neq \upsilon }{\sum }}‍\;\overset{n}{\underset{j=1,j<i,j=\upsilon }{\sum }}\frac{g_{ij}\left(\upsilon \right)}{g_{ij}}, \end{array}$$

where *g*_*ij*_ is the number of shortest paths between node *i* and node *j* and *g*_*ij*_(*υ*) is the number of shortest paths between node *i* and node *j* that pass through node *υ*.

An idea that a connection to a more interconnected node has more impact on the own centrality to a greater extent than a connection to a less well-interconnected node laid foundation of eigenvector centrality (EC). For a node *υ*, the EC is therefore given as [11]

(5) $$\begin{array}{c} \text{EC}\left(\upsilon \right)=\upsilon_{x}=\frac{1}{\lambda_{\max}\left(A\right)}\overset{n}{\underset{j=1}{\sum }}a_{jx}\upsilon_{j}, \end{array}$$

with *υ* = (*υ*_1_,*υ*_2_,…,*υ*_*n*_)^*T*^ referring to an eigenvector for the maximum eigenvalue *λ*_max⁡_(*A*) of the adjacency matrix *A*.

The other field as discussed earlier which is used in the proposed framework is outlier detection to predict any terrorist event. Outlier detection is very important because of the fact that outliers in the data point to something important that cannot be ignored. For example if extra ordinary traffic pattern is observed in a computer network, which obviously is an outlier, could indicate that a hacked computer is there in the network which may be sending important secret data outside the network. Such indication towards an event is possible due to outlier detection. Similarly, outlier transactions in credit card data could indicate that the card has been misused.

Some major causes of existence of outliers include [12] malicious activity: any activity that is exceptional and anomalous in a system, for example, credit card or telecom fraud, cyber intrusion, a terrorist activity, and instrument error. Some outliers may occur due to fault in components of measuring machines, environment change such as climate change, new buying pattern among consumers, mutation in genes, human error, data reporting error, and so forth.

Keeping in view the existing systems, we present a novel framework with two major contributions which are key player detection and prediction of any suspicious activity. All of the above centrality measures are important and provide valid information from different perspectives; so, selection among these can be a tough decision. To get full benefit and utilize strength of all of these, the proposed framework contains a classification based system of centrality to detect the key players using all of the above described measures. Secondly, the proposed model uses the idea of outliers which are created in the communication patterns of identified terrorist networks that something is likely to happen.

## 3. Proposed Model

**Figure 1:**
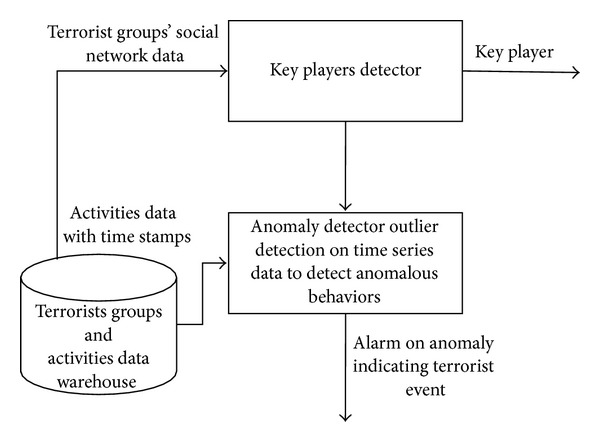
Flow diagram of proposed model.

The proposed model is based on the two contributions discussed earlier. So, aim of the model is to achieve two main objectives; the first is to find the key players of the terrorist networks which already have been detected and the second is to continuously monitor the activities of such groups with the aim to alarm the situations when the probability of a terrorist event is high.  Figure 1 shows the flow diagram of proposed model.

As mentioned earlier, the model consists of two major components; the first component is the key player detector which uses centrality measures and a hybrid classifier to detect all possible key players from the network. Once key players are detected, the data stream collected from different databases related to the actions that become the relationships of the network are monitored on time series in the second component of the model “anomaly detector” which has outlier detection in its core in order to predict terrorist activities. When an anomaly is detected that can be an indication of a severe terrorist activity like a suicide bomb attack or anything else, key players detected by the first component of the model can be eliminated by law enforcement agencies in order to destabilize the network and to prevent the event.

### 3.1. Data Preprocessing

The first step before applying proposed key player detection is data preprocessing. The purpose of this step is to clean the data in order to facilitate further steps. Data preprocessing consists of

- redundant feature removal,
- removal of duplicate entries,
- handling missing values.

**Figure 2:**
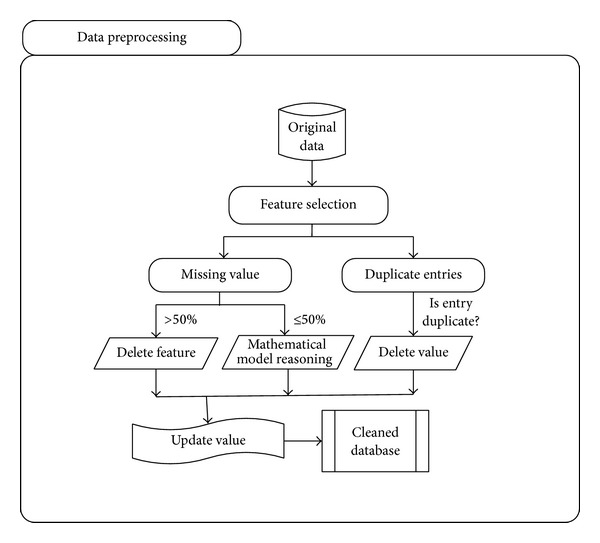
Flow diagram for handling data redundancy.

First step uses two rank sum tests, that is, Wilcoxon rank-sum and Ansari-Bradley tests. Wilcoxon rank-sum test is a nonparametric test of the null hypothesis that two populations are the same against an alternative hypothesis that the two distributions differ only with respect to the median. It has higher efficiency on nonnormal distributions such as a mixture of normal distributions [13]. Ansari-Bradley test compares two independent samples which come from the same distribution against the alternative that they come from the same distributions having the same median and shape but different variances [14]. Preprocessing step also checks for duplicate entries and removes all such entries to avoid redundancy. The last step in preprocessing is to handle missing values in the data. The preprocessing technique identifies the missing feature values and then they are replaced by the mean value for that feature. This procedure is performed for those attributes where values are missing in less than 50% of the instances. If the number of instances with missing values is more than or equal to 50%, the particular attribute is rejected and not used further.  Figure 2 shows the flow diagram of data preprocessing to handle any kind of data redundancies.

### 3.2. Key Player Detection

**Figure 3:**
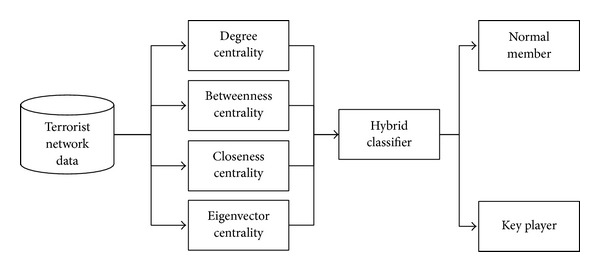
Proposed framework for key player detection.

Key player detection is an important step while analyzing covert networks. The proposed framework for key player detection consists of centrality measures for each node followed by hybrid classifier for accurate detection of key players. The four centrality measures which we have included in our proposed model are degree centrality (DC), betweenness centrality (BC), closeness centrality (CC), and eigenvector centrality (EC) which are given in (1), (4), (3), and (5), respectively.  Figure 3 shows the flow diagram of proposed model for key player detection.

Key players normally appear as most central nodes in any network; so, they have significant values of centrality measures. If a covert network *χ* contains *k* nodes, then the set representation for that network *χ* is *χ* = {*υ*_1_, *υ*_2_, *υ*_3_,…, *υ*_*k*_}. Here, *υ*_*i*_ represents *i*th node in given network. For an automated system to analyze each node as key player or normal player, a feature set is formed for each node. Each node is considered as sample for classification and represented by a feature vector containing four features; that is, for a sample node *υ* from a network *χ*, the feature vector is *υ* = {DC, BC, CC, EC}.

Once all nodes are represented by feature vectors, next phase is to classify them as key player or normal member. A new hybrid classifier as an ensemble of *k*-nearest neighbors (kNN), Gaussian mixture model (GMM), and support vector machine (SVM) is proposed here for accurate detection of key players. The purpose of using these three classifiers is to accurately model the distribution of data and to find accurate decision boundary by using the strengths of all three classifiers. kNN has simple implementation and gives good results whenever samples of same class exist as closest neighbors. GMM is famous due to the capability of accurately representing the data distribution and it caters for overlapping patterns where modeling of distribution gives a good clue. SVM caters for the data which is well separable by a decision boundary and has good classification and rapid training phase.

#### 3.2.1. *k*-Nearest Neighbors (kNN)

kNN is the most simple and fundamental classifier used for supervised classification [15]. It is a kind of voting based classifier which finds *k*-nearest samples from complete dataset based on some distance calculation between training and test samples. Let *υ*_*i*_ be a feature vector for *i*th node with *m* features (*f*_*i*1_, *f*_*i*2_, *f*_*i*3_,…, *f*_*im*_); let *n* be the total number of nodes (*i* = 1,2,…, *n*) and let *m* be the total number of features (*j* = 1,2,…, *m*). The Euclidean distance between nodes *υ*_*i*_ and *υ*_*l*_ where (*l* = 1,2,…, *n*) is defined as

(6) $$\begin{array}{c} d\left(x_{i},x_{l}\right)=\sqrt{{\left(x_{i1}-x_{l1}\right)}^{2}+{\left(x_{i2}-x_{l2}\right)}^{2}+\cdots +{\left(x_{ip}-x_{lp}\right)}^{2}}. \end{array}$$

Now, depending upon the value of *k*, we choose closest *k* samples and assign the majority class to unknown node.

#### 3.2.2. Gaussian Mixture Model (GMM)

To implement GMM, we use a two-class Bayesian classifier using Gaussian functions [16]. Bayes decision rule is stated as [17]

(7) Choose  R1  if,  p(v ∣ R1)P(R1)>p(v ∣ R2)P(R2) otherwise  choose  R2,

where *p*(**v**∣*R*_*i*_) is the class conditional probability density function (pdf) also known as likelihood and *P*(*R*_*i*_) is the prior probability of class *R*_*i*_ which is calculated as the ratio of class *R*_*i*_ samples in the training set. The class conditional pdf of the feature vector for different classes is computed using multivariate Gaussian pdf [17] as follows:

(8) $$\begin{array}{c} N\left(v\;|\;\mu ,\Sigma \right)=\frac{1}{{\left(2\pi \right)}^{m/2}{\left|\Sigma \right|}^{2}}\exp\left[\frac{-1}{2}\left(v-\mu \right)\Sigma^{-1}\left(v-\mu \right)\right], \end{array}$$

where **v** and *μ* are feature vector containing *m* number of features and mean vector containing mean of each feature, respectively. Σ is a *m* × *m* covariance matrix. In our case, *m* = 4. We model the class conditional pdf's as linear combination of weighted Gaussian functions to represent the likelihood of a GMM using (9) as follows:

(9) $$\begin{array}{c} p\left(v\;|\;R_{i}\right)=\overset{\kappa_{i}}{\underset{j=1}{\sum }}N\left(v\;|\;\mu_{j},\Sigma_{j}\right)\omega_{j}, \end{array}$$

where *κ*_*i*_ is the number of Gaussian mixtures used for Bayesian classification, *p*(**v**∣*R*_*i*_) is an *m*-dimensional Gaussian distribution of weight *ω*_*j*_, and *R*_*i*_ = {*R*_1_, *R*_2_} are the two classes used in proposed system. Equations (8) and (9) show the likelihoods for a single Gaussian distribution and GMM, respectively.

The parameters for GMM are optimized using expectation maximization (EM) which is an iterative method and it chooses optimal parameters by finding the local maximum value of GMM distributions for training data. The EM starts with initial values of parameters (*μ*, Σ) and weight *w* for each Gaussian. In estimation step, EM computes the probability (*P*_*E*_) of each point for each Gaussian using (10). One has

(10) $$\begin{array}{c} P_{E}\left(n,j\right)=\frac{w_{j}N\left(\upsilon_{n}\;|\;\mu_{j},\Sigma_{j}\right)}{\sum_{i=1}^{\kappa }N\left(\upsilon_{n}\;|\;\mu_{i},\Sigma_{i}\right)\omega_{i}}. \end{array}$$

Here, *P*_*E*_(*n*, *j*) represents the probability that *n*th candidate region *υ*_*n*_ is generated from *j*th Gaussian. We do this for all *κ* Gaussians and candidate regions. The second step is the maximization of likelihood by changing the parameters. The mean, covariance matrix, and weight for *j*th Gaussian are updated using estimated probabilities and are given in (11), (12), and (13), respectively, as follows:

(11) $$\begin{array}{c} \mu_{j}=\frac{1}{\xi_{j}}\overset{N_{\text{Total}}}{\underset{n=1}{\sum }}P_{E}\left(n,j\right)\upsilon_{n}, \end{array}$$

(12) $$\begin{array}{c} \Sigma_{j}=\frac{1}{\xi_{j}}\overset{N_{\text{Total}}}{\underset{n=1}{\sum }}P_{E}\left(n,j\right)\left(\upsilon_{n}-\mu_{j}\right){\left(\upsilon_{n}-\mu_{j}\right)}^{T}, \end{array}$$

(13) $$\begin{array}{c} \omega_{j}=\frac{\xi_{j}}{N_{\text{Total}}}, \end{array}$$

where *ξ*_*j*_ = ∑_*n*=1_^*N*_Total_^ *P*_*E*_(*n*, *j*) and *N*_Total_ are the total number of nodes.

#### 3.2.3. Support Vector Machine (SVM)

SVM is used as third classifier in proposed framework for key player detection. The original algorithm of SVM separates different regions from each other with maximum margin by using a separating hyperplane if the classes are linearly separable. Due to close relevance of nodes, the proposed features make a nonlinear hyperplane for which SVM is applied along with kernel function based on radial basis function (RBF). To implement SVM along with RBF, we have applied least squares SVM using LS-SVM toolbox [18]. In LS-SVM, the multiclass solution is found by solving a system of linear equations instead of original quadratic programming.

#### 3.2.4. Hybrid Classifier (HC)

**Figure 4:**
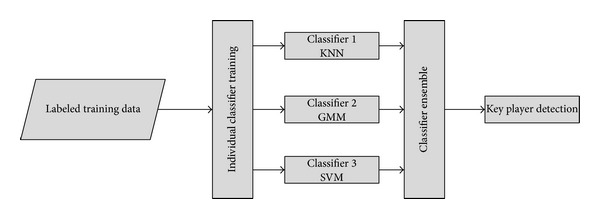
Proposed framework for hybrid classifier.

For hybrid classifier, we combine kNN, GMM, and SVM classifiers using a weighted probabilistic ensemble. The classification of node *υ* using probabilistic classification prediction, based on measure of evidence from different classifiers, is performed as

(14) $$\begin{array}{c} \text{class}\left(\upsilon \right)=\arg\underset{\forall \text{clas}{\text{s}}_{i}}{\max}\left(\overset{c}{\underset{i=1}{\sum }}a_{k}*P_{C_{k}}\left(y=\text{clas}{\text{s}}_{i}\;|\;\upsilon \right)\right), \end{array}$$

where *P*_**C**_*k*__(*y* = class_*i*_∣*υ*) is the probability of class_*i*_ given a sample node using classifier *k* and *a*_*k*_ is the weight associated with the probabilistic prediction of class **C**_*k*_.  Figure 4 shows the proposed ensemble framework for hybrid classifier.

*Learning Optimized Weights Using Genetic Algorithm.* The proposed ensemble framework given in (14) consists of a feature vector *a*_*k*_ = {*a*_kNN_, *a*_GMM_, *a*_SVM_}. These weights are optimized using genetic algorithm. The modeling of weights consists of two phases, that is, separation of confused samples and learning of optimized weights. In first phase, the algorithm separates out all confusing samples from complete training data. Confusing samples are those samples for which all three classifiers (kNN, GMM, and SVM) give different decisions and only these samples are used to optimize the weights for each classifier. This selection of confusing samples reduces the time for genetic algorithm in finding optimized weights. The second phase applies genetic algorithm for learning of optimal weights.

The parameters of genetic algorithm such as definition of population, size of population, rules for crossover and mutation, and objective function are defined as follows.

*(i) Population.* Each chromosome consists of a weight vector of three members which are weights for each classifier. All weight vectors are normalized to have a sum equal to 1.

*(ii) Population Size.* The initial population consists of 20 normalized weight vectors in which 16 are generated randomly and the remaining four are [1,0, 0], [0,1, 0], [0,0, 1], and [0.33,0.33,0.33]. Last four weight vectors are added to give maximum and equal confidence to all classifiers.

*(iii) Crossover.* Single point uniform crossover is used during learning. Crossover point is after first weight element which means that two selected chromosomes interchange their weights for GMM and SVM classifiers. The selection of chromosomes for crossover is performed based on objective function value. Worst 10 chromosomes out of population of 20 are selected for crossover.

*(iv) Mutation.* The mutation probability of 0% is used for mutation which means that no change is made in offsprings after crossover.

*(v) Objective Function.* The classification accuracy corresponding to a specific weight vector is taken as objective function as defined in (14) and we want to maximize this function.

The iterative learning is performed until there is no improvement in classification accuracy given in (14) for ten consecutive iterations or the algorithm reaches to maximum iteration which is set equal to 100.

### 3.3. Anomaly Detector

The second major component of the proposed model is the anomaly detector which has time series and outlier detection in its core. The motivation for this component is the concept of “prevention and prosecution” in combating terrorism. The intent is to continuously monitor the interactions between members of the terrorist group and to search for the outlier data depicting an extraordinary sort of activities pointing something is going to happen just like intrusion detection which is also an application of outlier detection.

The process of detecting anomalous observations from data is anomaly detection. In data, anomaly can arise due to different reasons like mechanical faults, other changes in the underlying system, fraudulent behavior, or any type of error. Generally, anomalous observations are more interesting because they can indicate a situation that needs to be dealt with. Same is in our scenario, where anomalous behavior of terrorist activists can be indicating a near future terrorist activity. As discussed earlier, anomaly detection is a widely researched area typically applied to various fields in order to detect any anomalous behavior like intrusion detection, fraud detection, and so on. Outlier detection has never been applied to predict a terrorist event monitoring terrorist activities data on a time series. So, the idea is novel which is proposed as second contribution in this paper.

The basic idea of anomaly detector is to detect anomalous activities of terrorist groups monitoring routine activities over a timeline and predict event whenever an outlier occurs. Suppose we have seven different databases which are integrated and which record the logs of activities of these members. An activity can be any action in which one actor performs an action on another actor or actors. For example, if a node “*A*” sends an SMS to node “*B*,” it will be recorded as activity in the SMS log database; actors are nodes “*A*” and “*B*” and the weight of activity is one because of single SMS. Similarly, if a node “*A*” sends an email to multiple nodes, the activity will be recorded in the email database as an activity and the weight of this activity will be equal to the total number of emails, that is, the total number of nodes receiving that email; say for example, if the email was sent to five nodes, the weight of overall activity will be equal to five. With these entire activities, time stamp will also be logged in the database in order to model activities on the timeline. At any instance of time, the overall weight of a terrorist group is proposed to be equal to sum of all the activities done by any member at that time. For example, if in our case we are considering SMS (*S*), telephonic conversation (TC), email (*E*), bank transfer (BT) of an extraordinary amount, and change of location (*L*), the aggregate weight of activities on any time instance will be equal to sum of numbers of all SMS sent at that time + sum of numbers of all emails exchanged at that time + sum of numbers of telephonic conversations + sum of numbers of bank transfers made + sum of numbers of change of locations as given in (15):

(15) $$\begin{array}{c} w\left(t\right)=\overset{n}{\underset{i=1}{\sum }}S_{i}+\overset{n}{\underset{i=1}{\sum }}\text{T}{\text{C}}_{i}+\overset{n}{\underset{i=1}{\sum }}E_{i}+\overset{n}{\underset{i=1}{\sum }}{\text{BT}}_{i}+\overset{n}{\underset{i=1}{\sum }}L_{i}. \end{array}$$

So, after taking aggregate sum of numbers of all the activities done at a time instance, the activities are modeled over a timeline. The next step is the continuous monitoring of the timeline in order to detect any outliers which can be an indication of a possible threat. The working of proposed anomaly detector consists of the following steps.

#### 3.3.1. Data Logging

All the activities of detected terrorist groups are logged in the corresponding datasets. Following is the glimpse of the sample databases used.

The data shown in all the databases depicts the activities performed at one time stamp. One time stamp can be any time interval, maybe an hour or a day.

#### 3.3.2. Integration

Data from all the sources is integrated into one central data warehouse that is used for central integrated information retrieval purpose.

#### 3.3.3. Aggregation

Aggregated summary of all the activities done on different time intervals is monitored on a time series. The input data is fetched from the central data warehouse. In the data shown in Tables 1, 2, 3, 4, and 5, the magnitude of activities generated by different nodes of a terrorist group on time stamp 1 is equal to 15 because, at this time, 3 SMS have been transferred, 3 emails have been transported, 3 bank transfers have been made, 3 telephonic conversations have been done, and 3 nodes have changed their locations; so, the aggregate sum is equal to 15.

The decision that this 15 is a normal or an outlier data object is made with the help of outlier detection. However, this is worth mentioning here that if 15 is a normal value, which is representing during one hour (we have taken time stamp equal to one hour), this terrorist group makes 3 SMS, 3 telephonic calls, 3 bank transfers, 3 emails, and 3 travels or may be near these values. This normal value shows that the group is in passive mode. Passive mode can be the preparation or planning phase of a terrorist group during which they may be preparing for an attack or may be making future strategy but if this value is an outlier, that is, if the number of activities performed during one hour is abnormal, the group may be going to carry out a terrorist attack; so, the law enforcement agencies should immediately eliminate the key players that key player detection component of the proposed model has already pointed out.

**Figure 5:**
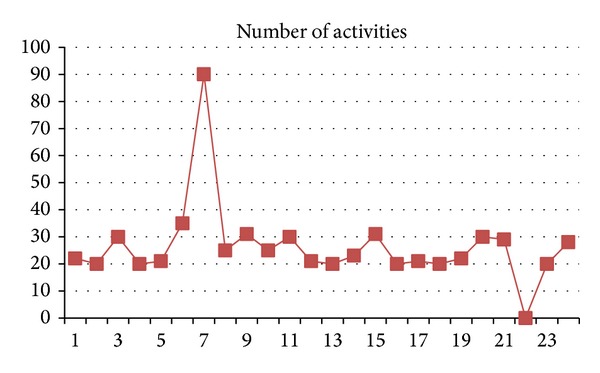
Activity monitoring over timeline.

In the graph shown in Figure 5, *x*-axis shows the time stamps, while magnitude of activities done by members of a group is shown along *y*-axis. Apparently, the activities done at time intervals 7 and 22 can be possible outliers indicating an indication of a terrorist attack but may be a false alarm.

Nearest neighbor analysis is a very well-known concept in which an object is analyzed with respect to its neighbors. For outlier detection in the proposed model, nearest neighbor analysis is proposed to be used to analyze the activities magnitude over time series because of its simplicity and because of its suitability for the data upon which we want to apply it to detect outliers. Nearest neighbor based outlier detection has the key advantage of being purely data driven.

**Figure 6:**
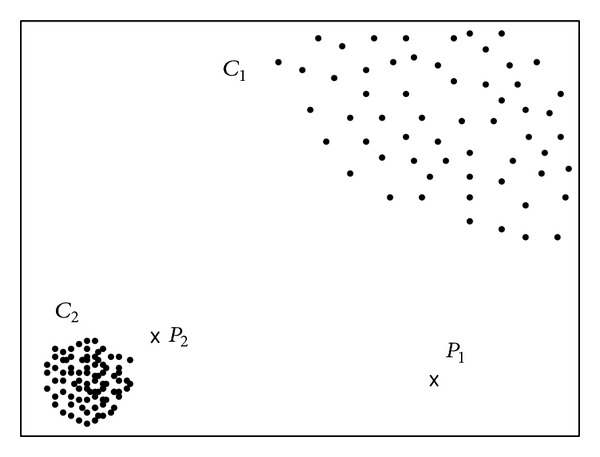
Nearest neighbor based approach.

As stated earlier, in nearest neighbor based outlier detection, the base assumption is that normal objects have many closely located neighbors, while outliers are located in a comparatively low dense region which is normally far from normal regions. As Figure 6 indicates, *C*_1_ and *C*_2_ are two data clusters in which all the data objects are closely located indicating that all are normal data instances, while points *P*_1_ and *P*_2_ are located in rare regions clearly depicting that they are outlier instances.

Nearest neighbor outlier detection techniques comprise of two steps; the first step is to compute neighborhood of each data object using a distance or a similarity measure defined between two data objects and then, in the second step, the neighborhood is analyzed to decide whether a data object is normal or outlier. Outlier detection techniques that fall under category of nearest neighbor operate using a distance or similarity measure which is defined between two data objects. There are different ways of computing distance or similarity between two data objects. Choice depends on the nature of data as follows.

- Euclidean distance is mostly used for continuous data attributes but other measures can also be used [19].
- Generally, a simple matching coefficient is used for data objects having categorical attributes. Some complex distance measures are also defined in [20, 21].
- Normally, distance or similarity is calculated for each attribute and then combined for multivariate data, that is, data with multiple attributes.

Broadly, nearest neighbor based techniques can be divided into two categories based on how they calculate the outlier score. The first category is “distance to *k*th nearest neighbor based.” In these techniques, distance of a data object to *k*th neighbor is calculated and is used as its outlier score. The second category is “relative density based.” In techniques belonging to this category, relative density of each data object is computed to get its outlier score. Choice depends on nature of data or any other priority. Distance based outlier score calculation is used in the proposed model. From neighborhood perspective, there are three well-known definitions of outliers as follows.

1. The data objects in a dataset have fewer than *k* neighbors where a neighbor is a data object that is within a distant *R* [22].
2. Data objects are the *n* objects presenting the highest distance values to their respective *k*th nearest neighbor [23].
3. Outliers are the *n* data objects in a dataset that present the highest average distance to their respective *k*-nearest neighbors [15].

The basic form of a *k*-nearest neighbor outlier detection is known as simple nested loops (SNL) algorithm which has worst case complexity *O*(*n* ^2^).  Algorithm 1 shows the outlier algorithm which we have used in our proposed system.

*Note. *Maxdist(*d*, *S*) returns the maximum distance between *d* and an element in set *S*. Closest(*d*, *S*, *k*) returns the *k*-nearest elements in *S* to *d*. TopOutlier(*S*, *n*) returns the top *n* outliers in *S* based on the distance to their *k*th nearest neighbor. MaxThreshold(*S*) returns the distance between the weakest outlier in *S* and its *k*th nearest neighbor.

## 4. Experimentation and Results

### 4.1. Material

An open source software NodeXL which plugs in with Microsoft excel is used for testing of key player detection. For proper evaluation of proposed framework, we have used three case studies. The nodes in all three networks are labeled as key players and normal members.  Table 6 shows the network specification for all three case studies on which the proposed system is evaluated.

#### 4.1.1. Case Study-I

**Figure 7:**
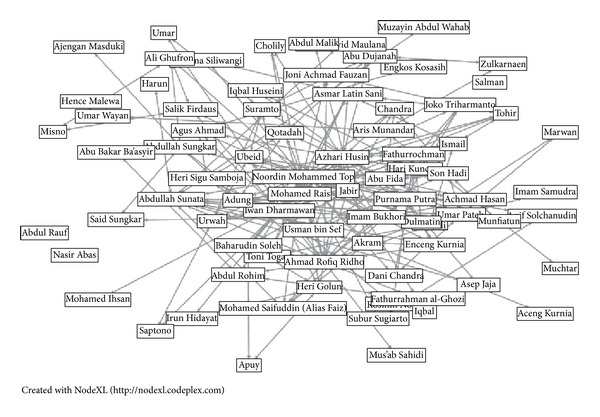
The Noordin Muhammad network.

The first case study is taken from [24] titled as Noordin Muhammad network. This subset of the Noordin Top Terrorist Network was drawn primarily from “Terrorism in Indonesia: Noordin's Networks,” a 2006 publication of the International Crisis Group. It includes relational data on the 79 individuals listed in Appendix C of that publication. The data were initially coded by Naval Postgraduate School students as part of the course “Tracking and Disrupting Dark Networks” under the direction of Professor Sean Everton, Codirector of the CORE Lab, and Professor Nancy Roberts. CORE Lab Research Associate Dan Cunningham also reviewed and helped clean the data.  Figure 7 shows network generated in NodeXL for Noordin's network.

#### 4.1.2. Case Study-II

**Figure 8:**
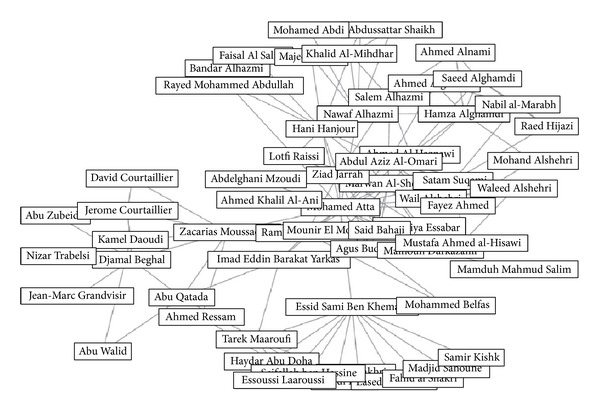
September 11 attackers network.

The dataset for second case study was first compiled by Krebs [1] consisting the tragic September 11 attackers network. The overall network consisted of 62 nodes and 150 edges containing all the attackers and their helpers who helped or coordinated in any way to organize the attacks. Muhammad Atta was the leader as confirmed by Ossama Bin Laden in a video tape [1]. The actual 19 hijackers who got crashed are labeled. They are considered important because they are the actual implementers of the attack.  Figure 8 shows network generated in NodeXL for September 11 attackers network.

#### 4.1.3. Case Study-III

**Figure 9:**
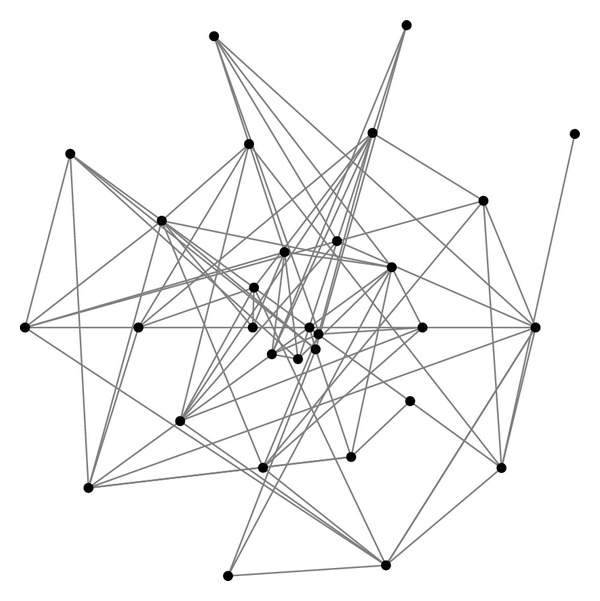
Cyber attackers network.

This dataset consists of real data that has been created by IT department of our institute during detection and capturing of a hackers group who were intruding in our institute's management information system. The network present in the dataset was created when a hacker was traced on a complaint; all his connections were traced from his communication links and his In/Out data log. The network consists of 30 nodes and 114 edges.  Figure 9 shows network generated in NodeXL for cyber attackers network.

### 4.2. Results

The detailed quantitative and comparative analysis of proposed system is performed in this section. The performance of proposed system is measured using sensitivity (sen), specificity (spec), accuracy (acc), and area under receiver operating characteristics (ROC) curves (AUC) as figures of merit. Sensitivity is true positive rate and specificity is true negative rate. These parameters are calculated using (16), (17), and (18), respectively, as follows:

(16) $$\begin{array}{c} \text{Sensitivity}=\frac{T_{P}}{\left(T_{P}+F_{N}\right)}, \end{array}$$

(17) $$\begin{array}{c} \text{Specificity}=\frac{T_{N}}{\left(T_{N}+F_{P}\right)}, \end{array}$$

(18) $$\begin{array}{c} \text{Accuracy}=\frac{\left(T_{P}+T_{N}\right)}{\left(T_{P}+T_{N}+F_{P}+F_{N}\right)}, \end{array}$$

where

- *T*_*P*_ are true positive mean numbers of key players which are identified correctly;
- *T*_*N*_ are true negative mean numbers of normal members of network which are identified correctly;
- *F*_*P*_ are false positive mean numbers of normal members of network which are wrongly identified as key players;
- *F*_*N*_ are false negative mean numbers of key players which are wrongly identified as normal members of network.

The modeling of classifiers has been done using randomly selected 70% of data as training and remaining 30% data as testing. The experiments are repeated 10 times and their average results are given.  Table 7 shows the results of proposed framework for key player detection on all three networks given in case studies.

**Figure 10:**
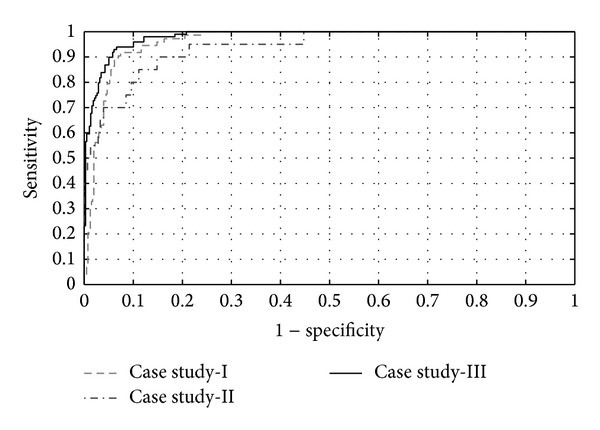
ROC curves of proposed framework for key player detection.

The statistical analysis of proposed system is done with the help of ROC curves which are plots of sensitivity versus 1-specificity. This analysis is done for performance evaluation of proposed hybrid classifier (HC).  Figure 10 shows the averaged ROC curves for all three case studies.

The proposed hybrid classifier is compared with individual kNN, GMM, and SVM classifiers. The hybrid classifier is also compared with the existing well-established classifier ensemble methods such as AdaBoost [25], bagging [26], and random subspace methods (RSM) [27].  Table 8 shows the comparison of all these in terms of accuracy for key player detection.

Table 9 shows the comparison of proposed hybrid classifier with existing state of the art classifiers such as multilayer perceptron (MLP), Bayes classifier, and logistic regression.

The third case study which is based on the local network is also used to evaluate anomaly detection component of proposed system. A campus In/Out system was used to monitor the In/Out activities of the group members. Applying the proposed outlier detection strategy, the group was captured right at the moment when they were ready to attack. Set of attributes consisting of the hour of day of in and out times was extracted from the dataset and outlier detection was applied. Results of only main key player are presented in Table 10 because of convenience as other individuals' outliers also appeared at the same time.

The points at which algorithm marked outliers are the points where there was a potential attack on the system. Out of three outliers detected in the log, that is, at S. numbers 4, 38, and 41, 4 and 38 were false alarms, while the group was captured on 41 where other correlations also identified as potential threat.

## 5. Conclusion and Detection

National security has attracted a number of researchers due to tragic events of terrorism across the globe especially from the field of information and communication technologies. This is because of the fact that, in order to carry out terrorist events, very extensive collaboration between terrorists can be observed including use of latest communication technologies. This opens antiterrorist activities research on the data collected from detected suspicious individuals.

Application of social network analysis has also become a very active area of research due to the above mentioned fact. In this paper, we have proposed a new framework for combating terrorism which consists of key player detection part which is from social network analysis and an event predictor part which has its base from outlier detection. For key player detection, in order to utilize existing most effective measures of key player detection, a novel hybrid classifier based system has been proposed which detected key players from given data. The second and important contribution is the prediction of any suspicious activity by monitoring the data of any network and identifying something abnormal by using time series analysis along with nearest neighbor analysis.

The proposed framework has been tested using three case studies and number of statistical measures. The results taken clearly prove the validity and correctness of proposed framework. A new study from actual local event is taken and proposed system is tested on that as well. Along with proposed framework for key player detection, we have also tested a voting based method which takes care of all four centrality measures to detect the top *k* key players of the terrorist network. Here, *k* is equal to the total number of key players present in a network which is under study. A node is considered as key player of three out of four centrality measures declared it as key player. This idea detected key players with accuracies of and 79%, 65%, and 83.33% for all three case studies, respectively. The proposed framework achieved accuracies of 91.52%, 88.73, and 95.91% for the same case studies, respectively. The results showed the validity of our framework and it can be used for detection of key players for any suspicious network along with detection of any abnormal activity.

## Figures and Tables

**Algorithm 1:**
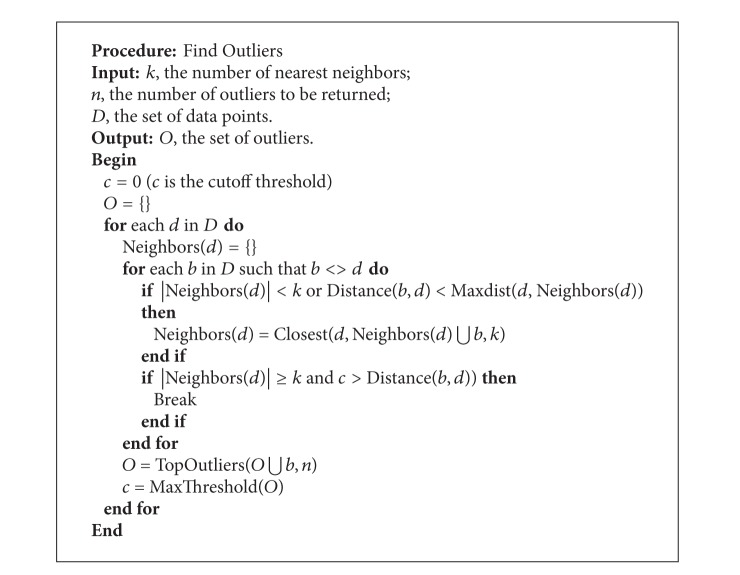
Algorithm to find outliers.

**Table 1:** SMS database.

Sender node ID	Receiver node ID	Time stamp	Text
1	2	1	Xyz
2	3	1	Xyz
2	4	1	Xyz

**Table 2:** Email database.

Sender node ID	Receiver node ID	Time stamp	Email
2	5	1	Some text
2	3	1	Some text
2	4	1	Some text

**Table 3:** Telephonic conversation database.

Caller node ID	Callee node ID	Time stamp	Duration
5	6	1	0:30:19
7	8	1	0:05:01
8	10	1	0:03:09

**Table 4:** Bank transfer database.

Transferring node ID	Transferee node ID	Time stamp	Amount
5	10	1	USD 10,000
10	11	1	USD 5,000
10	12	1	USD 7,000

**Table 5:** Travel record database.

Node ID	Source	Destination	Time stamp
12	sss	ddd	1
13	fff	ddd	1
14	Hhh	ddd	1

**Table 6:** Network specification.

Case study	Nodes	Edges	Key players
I	79	402	8
II	62	150	19
III	30	114	9

**Table 7:** Statistical performance evaluation of proposed framework for key player detection.

Case study	sen	spec	acc	AUC
I	93.69	89.71	91.52	0.91
II	89.31	87.03	88.73	0.86
III	95.03	96.42	95.91	0.93

**Table 8:** Comparison of hybrid classifier with existing ensemble methods.

Methods	Case study-I	Case study-II	Case study-III
KNN	84.35	80.09	81.32
GMM	87.72	84.38	87.50
SVM	88.03	81.25	93.75
AdaBoost	82.15	80.41	89.47
Bagging	86.78	81.57	90.72
RSM	86.13	82.61	87.03
Proposed hybrid classifier	**91.52**	**88.73**	**95.91**

**Table 9:** Comparison of proposed hybrid classifier with other states of the art classifiers.

Classifier	Case study-I	Case study-II	Case study-III
MLP	87.23	81.37	91.87
Bayes classifier	85.92	80.81	86.36
Logistic regression	86.39	82.49	90.74
Hybrid classifier	**91.52**	**88.73**	**95.91**

**Table 10:** Outlier detection results on cyber attackers network.

S. number	Hour of time out	Hour of time in	Result
1	7	15	Normal
2	13	15	Normal
3	18	16	Normal
4	1	13	Outlier
5	20	10	Normal
6	8	8	Normal
7	20	8	Normal
8	20	19	Normal
9	7	18	Normal
10	12	8	Normal
11	17	11	Normal
12	10	10	Normal
13	12	20	Normal
14	17	17	Normal
15	20	14	Normal
16	19	15	Normal
17	19	17	Normal
18	13	11	Normal
19	18	17	Normal
20	16	15	Normal
21	21	21	Normal
22	15	15	Normal
23	13	13	Normal
24	20	15	Normal
25	18	18	Normal
26	10	19	Normal
27	18	16	Normal
28	19	18	Normal
29	10	16	Normal
30	1	17	Normal
31	21	20	Normal
32	12	20	Normal
33	22	16	Normal
34	23	23	Normal
35	5	23	Normal
36	9	9	Normal
37	18	14	Normal
38	20	8	Outlier
39	18	18	Normal
40	23	17	Normal
41	23	23	Outlier
42	19	15	Normal
43	12	18	Normal
44	7	13	Normal
45	14	9	Normal
46	21	17	Normal
47	13	15	Normal
